# Angiotensin II Infusion Induces Marked Diaphragmatic Skeletal Muscle Atrophy

**DOI:** 10.1371/journal.pone.0030276

**Published:** 2012-01-20

**Authors:** Bashir M. Rezk, Tadashi Yoshida, Laura Semprun-Prieto, Yusuke Higashi, Sergiy Sukhanov, Patrice Delafontaine

**Affiliations:** Heart and Vascular Institute, Tulane University School of Medicine, New Orleans, Louisiana, United States of America; Northwestern University, United States of America

## Abstract

Advanced congestive heart failure (CHF) and chronic kidney disease (CKD) are characterized by increased angiotensin II (Ang II) levels and are often accompanied by significant skeletal muscle wasting that negatively impacts mortality and morbidity. Both CHF and CKD patients have respiratory muscle dysfunction, however the potential effects of Ang II on respiratory muscles are unknown. We investigated the effects of Ang II on diaphragm muscle in FVB mice. Ang II induced significant diaphragm muscle wasting (18.7±1.6% decrease in weight at one week) and reduction in fiber cross-sectional area. Expression of the E3 ubiquitin ligases atrogin-1 and muscle ring finger-1 (MuRF-1) and of the pro-apoptotic factor BAX was increased after 24 h of Ang II infusion (4.4±0.3 fold, 3.1±0.5 fold and 1.6±0.2 fold, respectively, compared to sham infused control) suggesting increased muscle protein degradation and apoptosis. In Ang II infused animals, there was significant regeneration of injured diaphragm muscles at 7 days as indicated by an increase in the number of myofibers with centralized nuclei and high expression of embryonic myosin heavy chain (E-MyHC, 11.2±3.3 fold increase) and of the satellite cell marker M-cadherin (59.2±22.2% increase). Furthermore, there was an increase in expression of insulin-like growth factor-1 (IGF-1, 1.8±0.3 fold increase) in Ang II infused diaphragm, suggesting the involvement of IGF-1 in diaphragm muscle regeneration. Bone-marrow transplantation experiments indicated that although there was recruitment of bone-marrow derived cells to the injured diaphragm in Ang II infused mice (267.0±74.6% increase), those cells did not express markers of muscle stem cells or regenerating myofibers. In conclusion, Ang II causes marked diaphragm muscle wasting, which may be important for the pathophysiology of respiratory muscle dysfunction and cachexia in conditions such as CHF and CKD.

## Introduction

Muscle wasting is associated with ageing and several chronic diseases including congestive heart failure (CHF) [1], [2], [3], [4], chronic kidney disease (CKD) [5], chronic obstructive pulmonary disease [6] and cancer [7]. Loss of lean body mass increases the mortality and morbidity of chronic disease states, e.g., it is a major predictor of poor outcome in CHF [8]. Muscle atrophy in chronic diseases is not restricted to limb muscles; it may also affect respiratory muscles which work continuously during life and have a higher ability to overcome fatigue compared to limb muscles, perhaps due to higher blood flow, capillary density, mitochondrial content, antioxidant capacity and oxygen consumption [9]. Thus respiratory muscle function and in particular function of the diaphragm is compromised in patients with chronic obstructive pulmonary disease [6], [9], CHF and CKD [2], [10].

The etiology of muscle wasting in chronic disease states is certainly multifactorial [11], and involves changes in physical activity, appetite and nutrient intake and increases in inflammatory mediators and cytokines such as TNF-α and IL-6 [12], [13]. It is of note that patients with CHF and CKD have stimulation of the renin-angiotensin system and often have high levels of circulating angiotensin II, even in the presence of angiotensin-converting enzyme inhibitor therapy [14], [15]. Studies from our laboratory have previously shown that Ang II infusion in rodents induced catabolic hind limb muscle wasting, secondary to multiple mechanisms including reduced insulin-like growth factor-1 (IGF-1) signaling, stimulation of the ubiquitin-proteasome pathway of protein degradation [16], [17] and increased skeletal muscle apoptosis. While these findings have suggested that Ang II could contribute to muscle wasting in chronic disease states the potential effect of Ang II on respiratory muscles remains unknown. This study was designed to investigate whether Ang II induced diaphragmatic muscle wasting and to obtain initial insights into mechanisms involved.

## Results

### Ang II induced diaphragmatic muscle wasting

**Figure 1:**
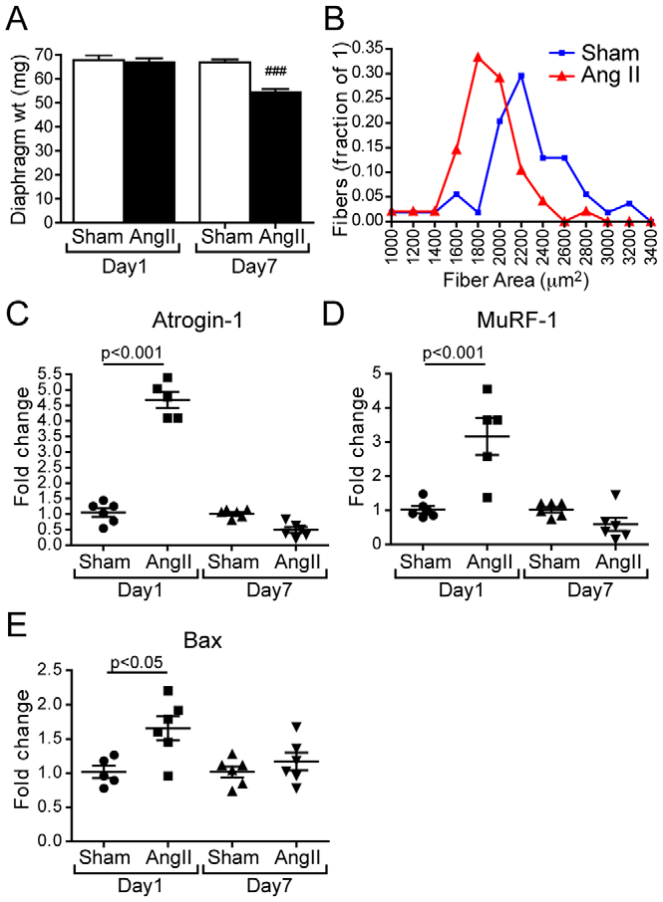
Diaphragm Muscle Wasting. (A) Diaphragm weight of sham at day 1 (Sham-D1), Ang II at day 1 (Ang II-D1), sham at day 7 (Sham-D7) and Ang II at day 7 (Ang II-D7). N = 8, Mean±SEM, ^###^P<0.001. (B) Cross sectional area of diaphragm at day 7 (Sham, blue; Ang II, red). (C–E) Quantitative RT-PCR of Atrogin-1 (C), MuRF-1 (D) and BAX (E).

To determine the potential effect of Ang II on diaphragmatic skeletal muscle, 9 week old FVB mice were subcutaneously infused with 1 µg/Kg/min of Ang II or sham-infused. As shown in Fig. 1A, Ang II induced significant diaphragmatic muscle wasting after 7 days, whereas Ang II had no significant effect at 24 h. Induction of diaphragmatic muscle atrophy was confirmed by a reduction in cross sectional area (CSA) of diaphragm myofibers (Fig. 1B, 26% leftward shift of myofiber area distribution curve). After 24 h of Ang II infusion, mRNA expression levels of the ubiquitin ligases atrogin-1 (Fig. 1C) and muscle ring finger-1 (MuRF-1) (Fig. 1D) were increased 4.6±0.3 and 3.1±0.5 fold, respectively, compared to sham infused mice, whereas there was no difference on day 7. Expression of the pro-apoptotic marker BAX was also elevated (1.6±0.2 fold) in response to Ang II infusion on day 1 (Fig. 1E).

### Regeneration of diaphragmatic skeletal muscle in response to Ang II-induced injury

**Figure 2:**
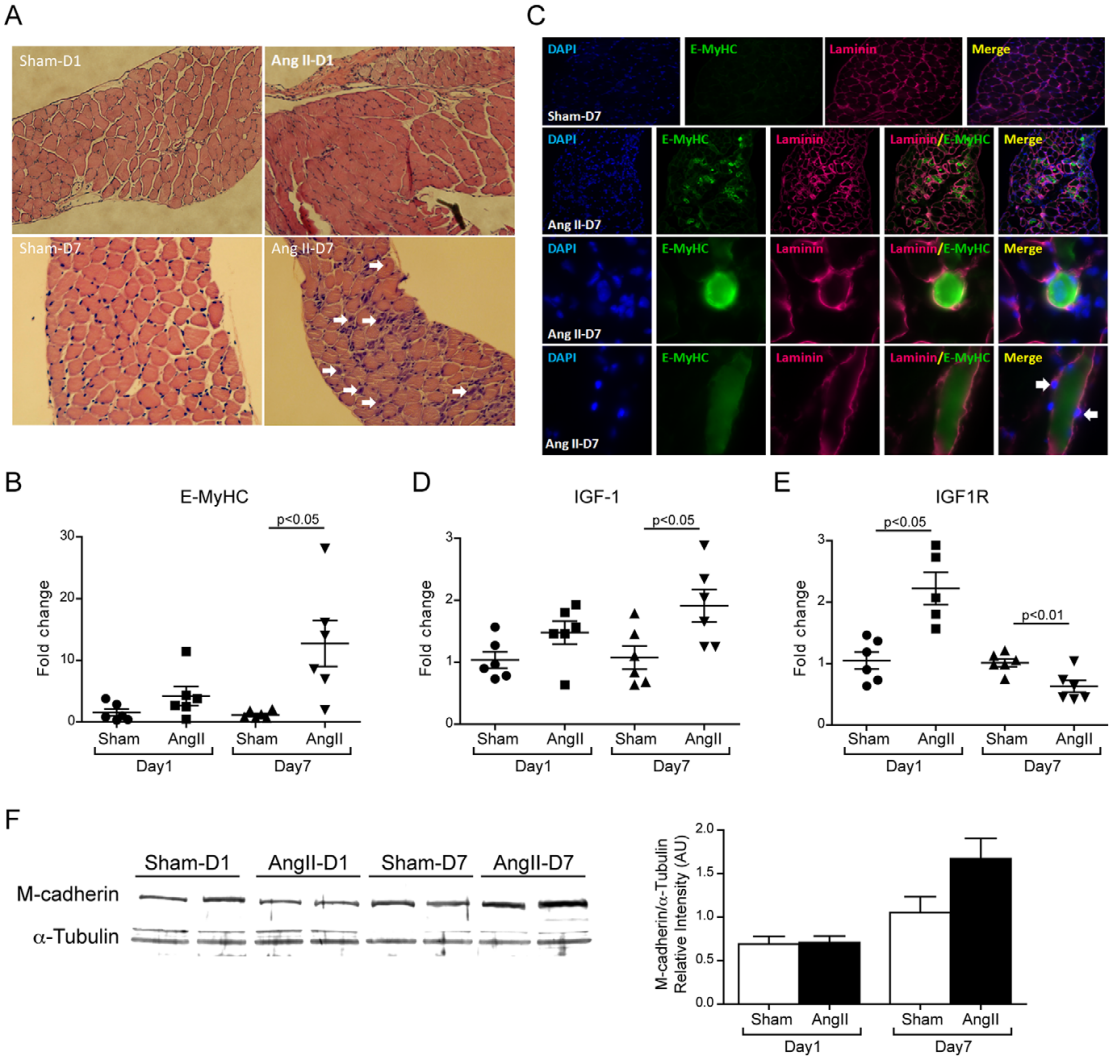
Regeneration of Diaphragm Muscles. (A) Representitive hematoxylen and Eosin staining sections at days 1 and 7 for sham and for Ang II. White arrows show centralized nuclei. (B) Quantitative RT-PCR of embryonic MyHC (E-MyHC). (C) Immunostaining of E-MyHC (green) and Laminin (magenta). Nuclei were stained with DAPI (blue). First row shows no expression of E-MyHC in the Sham infused mice at day 7(20× magnification). Diaphragm of Ang II infused mice (second row, 20× magnification) shows high expression of E-MyHC (green). Higher magnification (100×) of cross sections (third row) clearly shows the centralized nucleus in the newly formed myofiber. Fourth row represented the longitudinal section of a newly formed myofiber showing the location of satellite cells (whit arrows). (D, E) Quantitative RT-PCR of IGF-1 (D) and IGF-1R (E). (F) Immunoblot analysis showed a slight increase of M-cadherin in diaphragm of Ang II infused mice. N = 4, Mean±SEM, p = 0.08.

Hematoxylin and Eosin (H&E) stained sections showed centralized myonuclei at 7 days in Ang II infused animals (Fig. 2A), consistent with skeletal muscle regeneration. Quantitative PCR results indicated that expression of embryonic myosin heavy chain (E-MyHC) was increased 11.2±3.7 fold in diaphragm from Ang II infused mice compared to sham infused controls at one week (Fig. 2B). Immunohistochemical staining confirmed the increase of E-MyHC in the diaphragm of Ang II infused mice on day 7 (Fig. 2C). These E-MyHC positive myofibers were characterized by centralized nuclei (Fig. 2C, 3^rd^ row) and longitudinal sections (4^th^ row) indicated the location of satellite cells in the newly formed myofibers (white arrows). Insulin-like growth factor-1 (IGF-1) plays a permissive role in satellite cell activation and regeneration of skeletal muscle [18], [19], [20], [21]. Expression of IGF-1 in the diaphragm was significantly elevated after one week of Ang II infusion (Fig. 2D). The expression of IGF-1 receptor (IGF-1R) was significantly elevated at day one of Ang II infusion but then decreased at day 7 (Fig. 2E). Western blotting of diaphragm lysates demonstrated a 59.2±22.2% increase in expression of the activated satellite cell marker M-cadherin (N = 4, p = 0.08) after 7 days of Ang II infusion, consistent with diaphragm muscle regeneration (Fig. 2F).

### Recruitment of bone marrow derived cells

**Figure 3:**
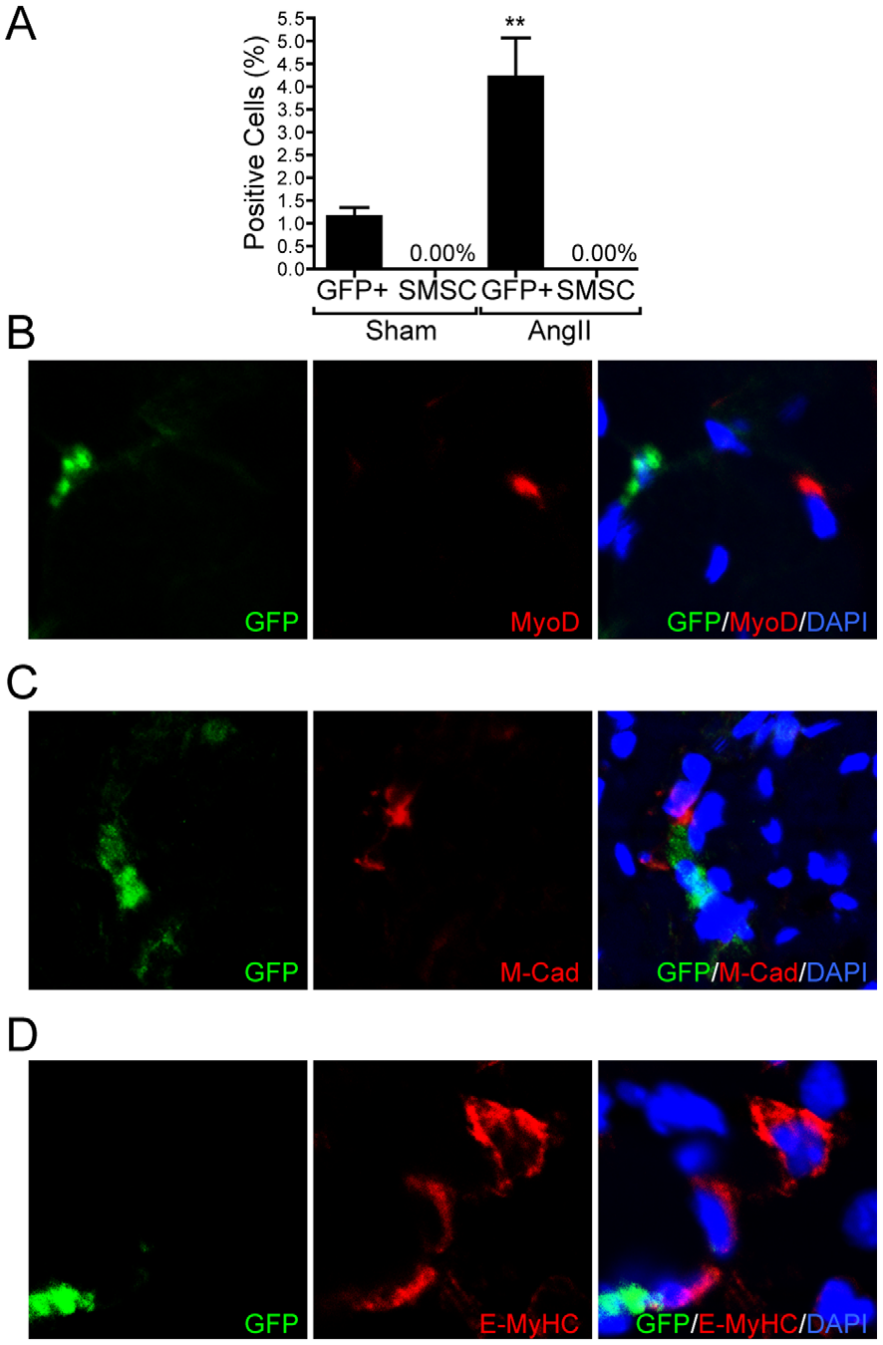
Recruitment of bone marrow derived cells. (A) Fluorescence activated cell sorter (FACS) analysis data at day 7 (Sham and Ang II infused mice) of recruited bone marrow derived cells (GFP^+^), and skeletal muscle stem cells (SMSC). N = 7, Mean±SEM, **P<0.01. (B–D) Bone marrow derived GFP positive cells recruited to diaphragm were costained with the satellite cell markers MyoD (B) and M-cadherin (C), and a marker of regenerating myofibers, E-MyHC (D).

Skeletal muscle injury induces inflammatory cell recruitment to the injured site to remove necrotic debris and to initiate the repair process [22], which involves activation, proliferation and differentiation of myogenic progenitor cells to repair or replace damaged muscle fibers. It has been shown that bone-marrow derived cells can be incorporated into injured skeletal muscle to form myotubes [23], although there is much evidence that muscle regeneration after injury involves predominantly local myogenic progenitor cells, called satellite cells [24], [25], [26]. To determine if Ang II-induced skeletal muscle injury and regeneration was associated with the recruitment of bone marrow derived muscle stem cells lethally irradiated mice were injected with bone marrow cells from GFP transgenic mice and infused with Ang II or saline three weeks after bone marrow transplantation. Our results indicated that bone marrow-derived GFP positive cells were significantly recruited into diaphragm of Ang II infused mice (Fig. 3A). However, none of the recruited cells were positive for the skeletal muscle stem cell (SMSC) markers Sca-1^−^/CD45^−^/CD31^−^/CD11b^−^/CD34^+^/Integrin-β1^+^/GFP^+^ [27], [28] (Fig 3A). Furthermore, none of these cells expressed the satellite cell markers MyoD or M-cadherin (Fig. 3B, C) or a marker of regenerating myofibers E-MyHC (Fig. 3D).

## Discussion

Ang II infusion for 7 days markedly reduced muscle weight and CSA of diaphragm myofibers (Fig. 1A, B), indicating that Ang II induced respiratory muscle atrophy. In previous studies we have shown that Ang II produced hind limb muscle wasting in rodents [16], [17], [29], [30], [31]. These studies demonstrated that the catabolic effect of Ang II was the result of alterations in several signaling pathways, namely reduced IGF-1/Akt signaling, increased caspase-3 activity, an increase in E3 ubiquitin ligase atrogin-1 and muscle ring finger-1 (MuRF-1) expression leading to increased activity of the ubiquitin-proteasome proteolytic pathway [32] and increased apoptosis of skeletal muscle. Consistent with our previous report on hind limb muscles, mRNA expression levels of atrogin-1, MuRF-1 and of the pro-apoptotic marker BAX were significantly elevated after 24 h of Ang II infusion (Fig. 1C, D and E), strongly suggesting that Ang II induced atrophy of the diaphragm also involved increased apoptosis and increased activation of the ubiquitin-proteasome pathway of protein degradation. We have previously shown a marked increase of caspase-3 activity and of ubiquitinated proteins in the skeletal muscle of Ang II infused mice [16]. However, contrary to what we have observed in hind-limb muscle (where we have detected very few regenerating myofibers after 7 days of Ang II infusion, data not shown), the diaphragm of Ang II infused animals showed significant muscle regeneration, as demonstrated by the presence of myofibers with centralized nuclei and increased expression of E-MyHC.

Skeletal muscle injury is normally accompanied by muscle regeneration, in order to repair or replace damaged muscle fibers. For instance, in Duchenne muscular dystrophy there is ongoing regeneration in the setting of muscle degeneration and atrophy, although this regeneration is insufficient to match the pace of degeneration [33]. Muscle regeneration is characterized by recruitment of inflammatory cells and bone marrow-derived progenitor cells, activation and proliferation of muscle stem cells called satellite cells, differentiation of myoblasts and subsequent fusion to form immature multinucleated myofibers with centralized nuclei, often expressing E-MyHC [34], [35]. Our data clearly indicated evidence of regeneration in diaphragm of Ang II infused animals, namely centrally located nuclei (Fig. 2A), increased expression of E-MyHC (Fig. 2B and C) and a strong trend to increased expression of M-cadherin, a marker of activated satellite cells [36], [37] (Fig. 2F). IGF-1, acting via the IGF-1R, is an important regulator of skeletal muscle growth and regeneration [38], [39]. We found that expression of IGF-1 was significantly elevated in the diaphragm after one week of Ang II infusion (Fig. 2D), whereas we have previously shown that IGF-1 expression is reduced by Ang II in hind-limb muscle [16], [17]. Furthermore, Ang II increased IGF-1R expression in diaphragm at 1 day (Fig. 2E), consistent with the known effect of Ang II to transcriptionally regulate IGF-1R expression in vascular smooth muscle [40], [41]. Of note, at 7 days we observed that IGF-1R mRNA expression in the diaphragm was reduced in Ang II infused animals, consistent with the known ability of IGF-1 to downregulate expression of its receptor in other tissues [42]. Taken together our findings suggest that IGF-1 may play a role in regenerative processes in the diaphragm in the setting of Ang II induced injury.

To determine whether muscle regeneration in the setting of Ang II-induced wasting involved recruitment of bone marrow derived cells, we transplanted bone marrow cells from GFP-transgenic mice into age matched irradiated recipient mice, followed by Ang II infusion. Our results indicated that GFP positive cells were significantly recruited into Ang II injured diaphragm (Fig. 3A). These findings are in agreement with the studies of LaBarge and Blau [43], Camargo et al. [44], Musarò et al. [45] and Sherwood et al. [27] which reported that bone marrow derived cells were recruited into injured muscles. However the contribution of bone marrow derived cells to myogenesis and regeneration is likely not direct since these cells have minimal capacity to differentiate to myotubes [27], [46]. Under our experimental conditions, none of the recruited cells were positive for the satellite cell markers Sca-1^−^/CD45^−^/CD31^−^/CD11b^−^/CD34^+^/Integrin-β1^+^. These markers together with CXCR4^+^ were used by Sherwood *et al.* [27], to identify myogenic projenitor cells and in our analysis CXCR4^+^ was omitted to include GFP. Furthermore, we did not observe GFP^+^ myotubes in our Ang II infused mice, and none of the GFP positive cells expressed the satellite cell markers MyoD or M-cadherin or the marker of regenerating myofibers, E-MyHC (Fig. 3B–D).

Our finding that Ang II causes marked diaphragmatic muscle atrophy has major clinical implications. Conditions such as CHF and CKD are often characterized by increased circulating Ang II levels and the dose of Ang II used in our study produces a 2.8 fold increase in plasma Ang II [47], consistent with the increase found in patients with CHF and CKD [14], [15], [48], [49]. Both these conditions are associated with significant respiratory muscle dysfunction [2], [10] and mechanisms are poorly understood, although in CHF an increase in oxidant stress and in TNF-α may play a role [50]. Patients with chronic obstructive pulmonary disease also have respiratory muscle dysfunction which, in addition to mechanical causes, has been linked to an increase in atrogin-1 and activated caspase-3 expression and to increased activity of the ubiquitin-proteasome pathway[6], [51]. While there is little information on activity of the renin-angiotensin system in patients with chronic obstructive pulmonary disease [52], [53] one could speculate that Ang II contributes to these changes. Furthermore, McClung *et al.* [54] reported that active caspase-3 protein expression was upregulated in diaphragm muscle during mechanical ventilation-induced atrophy, and we have previously demonstrated that Ang II induces caspase-3 activation in skeletal muscle [16].

In summary, our data indicate that Ang II infusion in FVB mice causes marked diaphragm muscle atrophy. Expression of the E3 ligases atrogin-1 and MuRF-1 and of the pro-apoptotic factor BAX is increased by Ang II, consistent with increased proteolysis and apoptosis. In addition there is evidence of regenerating myofibers in the atrophied diaphragm and evidence of recruitment of bone-marrow derived cells, although these cells do not express muscle stem cell markers. Since the diaphragm plays a critical role in respiration, our findings may be important for understanding mechanisms of respiratory muscle dysfunction in chronic diseases such as CHF and CKD in which the renin-angiotensin system is activated.

## Materials and Methods

### Animals

All the animal experiments were approved by the Institutional Animal Care and Use Committee at Tulane University. Male FVB mice (9 weeks) were obtained from Charles River and were housed at the Tulane University animal care facilities under conventional conditions with constant temperature and humidity and fed a standard diet. FVB mice were subcutaneously infused with Ang II (1 µg/kg/min) or sham infused using ALZET osmotic minipumps. After one day and one week animals were sacrificed using ketamine/xylazine and diaphragms were harvested.

### Histology and Immunostaining

Diaphragm was fixed in 10% zinc formalin and paraffin-embedded. Paraffin sections were processed for Hematoxylin and Eosin stains to evaluate centrally located myonuclei. The cross-sectional area (CSA) of diaphragm muscle fibers was calculated by determination of the circumference of ∼40 adjacent cells from every muscle section examined (n = 10 sections/5 mice). The image was analyzed using Image Pro Plus (Media Cybernetics). For immunohistochemical staining, sections were fixed with 4% paraformaldehyde and permeabilized with 0.2% Triton X-100. After blocking, sections were incubated with primary antibodies overnight at 4°C. Antibodies used in this study are E-MyHC (1∶100; Developmental Studies Hybridoma bank), Laminin (1∶500; R&D Biosystems), MyoD (1∶50; DAKO) and M-cadherin (1∶100; BD Pharmingen). Alexa Fluor 488 or 594-conjugated secondary antibodies (1∶500: Invitrogen) were used for detection. Sections were analyzed under Olympus 1X81 Fluorescence Microscopy or Leica Confocal Laser Scanning Microscopy.

### Quantitative real-time RT-PCR

Diaphragm muscles were homogenized in Tripure isolation reagent (Roche), and total RNA was isolated with RNeasy Mini Kit (Qiagen). cDNA synthesis was performed using the RT^2^ first-strand kit (SABiosciences). Quantitative real-time PCR was performed using a 40-cycle two-step PCR protocol in the iCycler IQ real-time detection system (Bio-Rad). Hprt1 gene expression was used as an internal control. PCR primers used in this study were obtained from SABiosciences.

### Immunoblotting

The tissue lysate (25 µg protein) were subjected to sodium dodecyl sulfate polyacrylamide gel electrophoresis (SDS-PAGE) and transferred to a nitrocellulose membrane (Amersham Pharmacia). The membrane was blocked and incubated with an antibody against M-cadherin (Abcam) overnight at 4°C. The membrane was then incubated with the horseradish peroxidase-conjugated anti-rabbi IgG (Amersham Pharmacia) at a 1∶ 2000 dilution for 1 h at room temperature. The membrane was then processed using enhanced chemiluminescence (ECL) western blot detection reagent (Amersham Pharmacia).

### Bone marrow transplantation

Recipient FVB mice (6 weeks) received lethal gamma irradiation (900 rad). After 4–6 hours, recipient mice received 9×10^6^ GFP^+^ bone marrow cells isolated from the femur and tibia of age matched enhanced green fluorescent protein (EGFP) transgenic mice strain that expresses EGFP driven by chicken beta-actin promoter and CMV immediate early enhancer (FVB.Cg-Tg(ACTB-EGFP)B5Nagy/J, Jackson Laboratory). After three weeks recipient mice were infused with Ang II or sham infused for an additional week. Mice were then euthanized and perfused with normoosmotic saline. The efficiency of bone marrow transplantation was 88%, which was tested by quantitating GFP^+^ cells in femur of recipient mice. Diaphragm muscles were dissected and mononucleated cells were isolated for FACS analysis as described in the following section.

### FACS analysis

Diaphragm muscles were dissected and digested with 100 U/ml of collagenase type II (Worthington Biomedical Corporation) for 45 min and then 1.25 mg/ml of pronase (Roche) for 45 min at 37°C. Mononucleated diaphragm cells were filtrated with cell strainer (70 µm) (BD Falcon) and then incubated with primary antibodies (CD45, CD31, Sca-1, and CD11b, PE; CD34, APC; Integrin-β1, PE-Cy7. BD pharmingen) for 20 min at room temperature in the dark. Negative controls of diaphragm cells were incubated with equivalent amount of isotype IgG. All samples were washed three times by PBS pH 7.4. Flow cytometry analysis was performed by FACSVantage (Beckman Coulter).

### Statistical analysis

All data were expressed as mean ± SEM and analyzed using Student's *t*-test (Sham D1 *vs* Ang II D1 and Sham D7 *vs* Ang II D7). A p-value of <0.05 was considered significant.
